# Expiratory time constant patterns reveal obstructive lung mechanics in non-obstructive spirometry

**DOI:** 10.1371/journal.pdig.0001653

**Published:** 2026-08-11

**Authors:** Filip Depta, Surya P. Bhatt, Manuel Heinz, Radek Černý, Vladimír Koblížek, Pavol Pobeha

**Affiliations:** 1 Pavol Jozef Šafárik University, Medical Faculty, Košice, Slovakia; 2 Department of Critical Care, East Slovak Institute for Cardiovascular Diseases, Košice, Slovakia; 3 Center for Lung Analytics and Imaging Research (CLAIR) and Division of Pulmonary, Allergy, and Critical Care Medicine, University of Alabama at Birmingham, Birmingham, Alabama, United States of America; 4 MedTech and Software Development, Bad Kissingen, Germany; 5 MR Diagnostic, Prague, Czech Republic; 6 Department of Pulmonary Medicine, University Hospital Hradec Králové, Hradec Králové, Czech Republic; 7 Department of Respiratory Medicine and Tuberculosis, Louis Pasteur University Hospita l, Košice, Slovakia; Shanghai United Imaging Intelligence Co Ltd, CHINA

## Abstract

Based on the principle that increased airway resistance prolongs the expiratory time constant (TC), we investigated whether TC patterns could serve as a signature for obstruction. In this cross-sectional multicenter study of 19,937 validated spirometry records, we divided the descending expiratory flow-volume curve into five equal volume segments and derived consecutive TCs for each. A K-means algorithm trained on the normal subset defined data-driven patterns, which were then used to classify the entire cohort. We quantified pattern prevalence and used propensity score matching to assess spirometric differences between patterns within each spirometric category. Two distinct patterns were identified: a ‘normal’ pattern (stable consecutive TCs) and an ‘obstructive’ pattern (progressively prolonging TCs). The obstructive pattern was present in 7% of normal spirometry, 13% of possible restriction, and 33% of nonspecific FEV_1_ reduction, while 9% of patients meeting obstruction criteria had a normal TC pattern. In normal spirometry group, the obstructive pattern group showed lower forced expiratory volume in 1 second (FEV_1_) (males: 2.7 vs 3.0 L; females: 1.8 vs 2.0 L), reduced FEV_1_/forced vital capacity (FVC) (males: 67% vs 76%; females: 68% vs 78%), and impaired small airway metrics, including maximal mid-expiratory flow (MMEF_25–75_) (males: 1.6 vs 2.6 L/s; females: 1.0 vs 1.8 L/s) and FEV_3_/FEV_6_ (males: 91% vs 94%; females: 91% vs 95%), all p < 0.001. Other spirometric groups mirrored these findings. The obstructive TC pattern represents a distinct physiological phenotype suggestive of obstructive lung mechanics and is highly prevalent across common spirometric categories.

## Author summary

Spirometry, a routine breathing test, is the cornerstone of diagnosing common respiratory diseases such as asthma or chronic obstructive pulmonary disease, but its standard measures can miss early or mild airway narrowing. We explored a complementary way to read the same test by dividing the exhaled breath into five equal-volume segments and measuring how quickly the lungs empty in each. This speed of lung emptying is called the expiratory time constant. In healthy lungs, these time constants stay roughly stable across the breath because lungs are not affected by disease; when the airways are obstructed, each successive segment empties more slowly, producing a progressively time constant prolongation. Using machine learning, we identified a normal (stable) pattern and an obstructive (rising) pattern. The obstructive pattern appeared in roughly one in ten people whose conventional spirometry was classified as normal, and these individuals had consistently worse spirometry, mainly those parameters associated with small-airway function. Our findings suggest that reading the spirometry curve through time constants can reveal hidden obstructive lung mechanics, potentially helping detect lung disease earlier.

## Introduction

Obstructive lung diseases, including chronic obstructive pulmonary disease (COPD), are a leading cause of global morbidity and mortality [1]. COPD, characterized by irreversible airflow limitation, affects over 10% of the world’s population and is the third leading cause of death, creating a significant economic burden with direct healthcare costs approaching €40 billion in the European Union annually [2,3].

A persistent challenge in clinical practice is diagnosing early and mild airflow obstruction largely because conventional spirometric metrics lack sufficient sensitivity [4]. The primary diagnostic tool, the ratio of FEV_1_ (Forced Expiratory Volume in one second) to FVC (Forced Vital Capacity), is confounded by demographics [5]. While age-adjusted z-scores from the GLI (Global Lung Function Initiative) mitigate this issue, their reliance on a single, static threshold can still delay the detection of mild small airway disease, particularly subtle changes in the peripheral airways that often precede a significant decline in FEV_1_ [6,7].

Other conventional markers provide complementary information but carry trade-offs. The Maximal Mid-Expiratory Flow (MMEF_25–75_) reflects small-airway function and correlates with CT-defined small-airway disease; in the multinational Burden of Obstructive Lung Disease (BOLD) study, an isolated reduction below the LLN predicted future chronic airflow obstruction and outperformed the FEV_3_/FVC ratio [7,8]. Its routine use is nonetheless limited by wide variability and FVC dependence [9–11]. FEV_3_-based ratios (FEV_3_/FVC, FEV_3_/FEV_6_) are likewise practical surrogates for early airway disease but lose sensitivity in borderline cases [12,13]. Even the FEV_1_/FVC threshold is debated, with data-driven work suggesting an optimal cut-off nearer the 9th–10th percentile rather than the fixed lower limit of normal (LLN, z-score −1.645) [14]. Furthermore, a dissociation exists when the shape of the flow-volume ($\dot{\text{V}˙}$/V) curve visually suggests obstruction, yet the derived spirometric values remain above the LLN. This conflict creates considerable diagnostic uncertainty, as visual patterns have been linked to worse symptoms and underlying emphysema [15,16].

The expiratory time constant (TC) offers a physiological alternative. Although historically a domain of mechanical ventilation, TC is universally defined as the product of airway resistance (Raw) and respiratory system compliance (Crs). This relationship can be mathematically simplified to a ratio of exhaled volume to flow. For spirometry, TC is estimated by substituting the FVC for volume and the Peak Expiratory Flow (PEF) for flow, creating a ratio that numerically describes the $\dot{\text{V}˙}$/V curve [17,18]. While TC seems to be useful in detecting airflow obstruction earlier than the FEV_1_/FVC, a single TC value provides only a rough assessment of overall lung mechanics and can mask significant regional differences [19].

To overcome this limitation, a more granular analysis of compartmental TCs is possible using a ‘slicing’ technique, where the $\dot{\text{V}˙}$/V curve is divided into equal volume compartments, as originally proposed by Guttmann et al. [20] This method transforms the often heterogeneous shape of the $\dot{\text{V}˙}$/V curve into a series of TCs, with the underlying principle being that an increase in Raw causes each sequential TC to increase [21]. This creates a distinct ‘obstructive pattern’ - a signature of obstructive lung mechanics - characterized by a series of consecutively prolonged TCs.

Building on this physiological rationale, our study aimed to apply the ‘slicing’ method to the descending portion of the $\dot{\text{V}˙}$/V curve to determine the prevalence of the obstructive pattern across the common spirometric categories [22]. Additionally, we aimed to characterize the distinct spirometric profiles associated with each physiological pattern.

## Materials and methods

### Ethics statement

The study was approved by the Independent Ethics Committee of Pavol Jozef Šafárik University, Košice, followed by each institution’s Institutional Review Boards. Owing to the retrospective design, the requirement for informed consent was waived.

### Study population and spirometry

**Fig 1:**
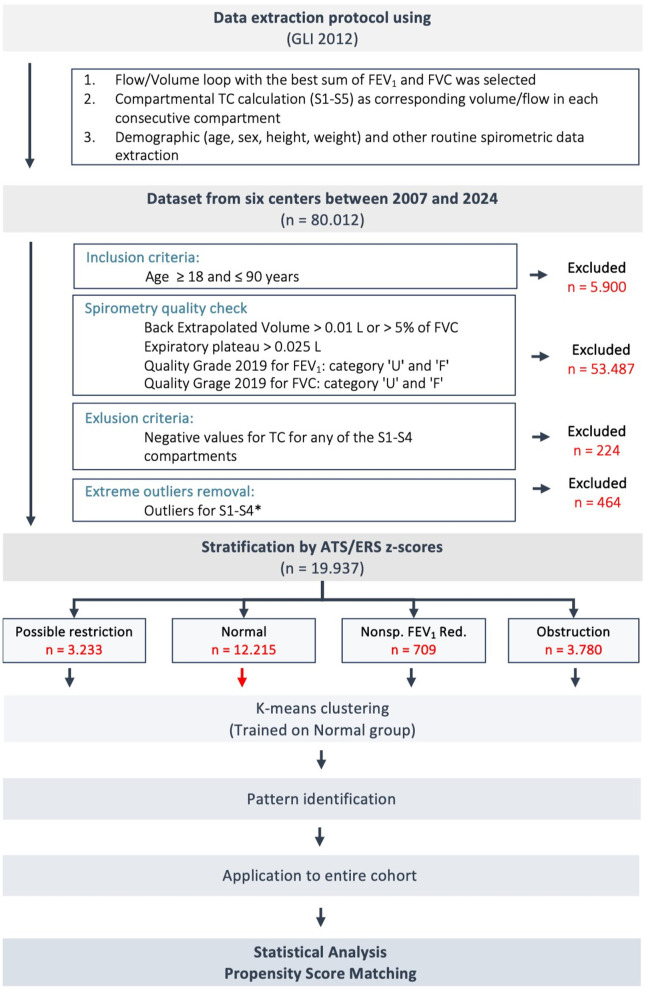
Study Flowchart and Data Processing. The initial dataset of 80,012 records was reduced to a final cohort of 19,937 after applying exclusion criteria for age, poor spirometry quality, negative compartmental values (S1-S4), and extreme statistical outliers (defined as values exceeding 8 × the Mean Absolute Deviation, or MAD). The final cohort was then stratified by 2022 ATS/ERS criteria and subjected to unsupervised machine learning (K-means clustering) for automatic TC pattern identification. Abbreviations: ATS/ERS, American Thoracic Society/European Respiratory Society; FEV1, Forced Expiratory Volume in 1 second; FVC, Forced Vital Capacity; MAD, Mean Absolute Deviation; S1–S4, first four compartmental expiratory time constants. Note: Extreme values were removed using an 8 × MAD threshold to minimize boundary artifacts before K-means clustering. This cutoff was selected as a robust, conservative filter for the heavy-tailed distribution of TC values.

Following approval from the Independent Ethics Committee of Pavol Jozef Šafárik University and Institutional Review Boards at each participating institution, we assembled a retrospective dataset comprising 80,012 pre-bronchodilator spirometric records collected at six high-volume centers in Slovakia and the Czech Republic. From an initial dataset of 80,012 records, we applied stepwise filtering to establish a final cohort of 19,937 subjects. Inclusion was restricted to adults (18–90 years) with high-quality spirometry meeting ATS/ERS criteria, after excluding technical artifacts (negative TC values) and extreme outliers (Fig 1) [10]. We selected only the first spirometry session for each patient to avoid data duplication, even if multiple records existed. An automated algorithm then processed these records to calculate compartmental and global TCs while simultaneously extracting demographic and standard spirometric variables [23].

### Interpretation of spirometry results

Using GLI-2012 reference equations and a LLN defined as a z-score of −1.645, the final cohort was stratified into four broad spirometric categories: (1) Normal: with FEV_1_, FVC, and FEV_1_/FVC all > LLN; (2) Possible restriction: FVC < LLN and FEV_1_/FVC > LLN (FEV_1_ may be above or below LLN); (3) Nonspecific FEV_1_ reduction: with FEV_1_ < LLN and both FVC and FEV_1_/FVC > LLN; and (4) Obstruction: with FEV_1_/FVC < LLN (FEV_1_ and FVC may be above or below LLN) [22].

### Derivation of compartmental time constants

For each patient, the flow-volume ($\dot{\text{V}˙}$/V) curve with the highest sum of FEV1 and FVC was selected for analysis. The descending expiratory portion of the $\dot{\text{V}˙}$/V curve, extending from peak expiratory flow (PEF) to the end of exhalation, was divided into five equal-volume compartments following the methodology described by Guttmann [20]. This compartmental approach was selected to align with established physiological models investigating compartmental lung emptying [24].

**Fig 2:**
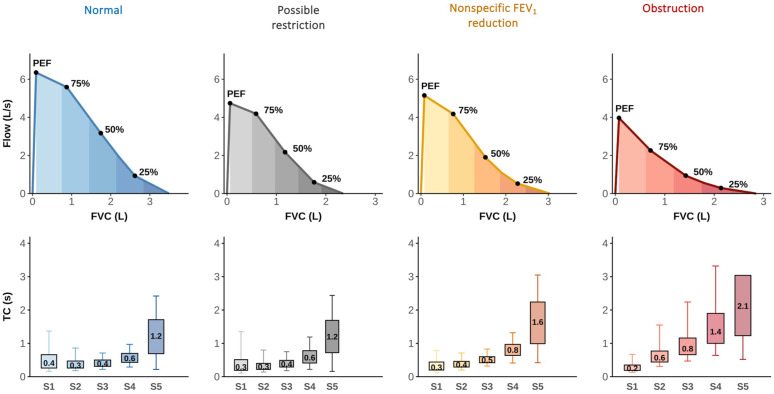
Spirometric Patterns and Analysis of Compartmental Time Constants. This figure presents average reconstructed spirometric flow/volume loops derived from the analysis of 19,937 as per the ATS/ERS criteria. Descending portion of the flow/volume loops was divided into five equal volume compartments using the slicing method, and TC (S1–S5) was calculated for each. The bottom plot shows boxplots with the median (IQR) TC values for each compartment. A distinguishing feature is the relatively homogenous time constants (S1 to S4) across compartments in the normal pattern, in contrast to the clear ascending trend present in patients with obstruction. Percentages correspond to Maximum Expiratory Flows are 75%, 50%, and 25% of FVC. Abbreviations: FEV1, Forced Expiratory Volume in 1 second; FVC, Forced Vital Capacity; TC, Time Constant; S1–S5, compartmental expiratory time constants.

Then, five consecutive TCs were calculated for each compartment labeled S1 (first compartment) through S5 (last compartment), and were obtained as the ratio of each compartmental volume to its corresponding flow decay. This decay was determined by the best fit of a regression line applied to the flow-time data. This process effectively transformed the spirometric curve into a sequence of five consecutive numerical TCs (Fig 2). Additionally, the duration of each iso-volumetric compartment was measured. A summary of the compartmental volumes, expiratory times, and calculated TCs is provided in S1 Table.

### Unsupervised clustering and pattern identification

To objectively classify patterns of lung emptying, an unsupervised machine learning (K-means clustering algorithm) was employed [25]. The primary predictors were the first four expiratory TCs and their corresponding durations. The TCs characterized the physiological airflow dynamics (ratio of volume and corresponding flow in each compartment), while the measured duration of each compartment provided the directly measured time component.

To establish an unbiased baseline of normal expiratory mechanics, the K-means model was trained on the subgroup classified as having normal spirometry by the LLN (*N* = 12,215). This approach defined a physiological reference for lung emptying mostly uninfluenced by disease, allowing the obstructive pattern to be identified as a deviation from normal mechanics rather than a pattern shaped by the heterogeneous pathologies present in non-normal spirometric categories. The final compartment (S5) was excluded from the model because 8% of subjects exhibited negative TC values, likely due to measurement artifacts caused by low signal quality at terminal flow rates. Preliminary testing confirmed that including S5 did not improve model performance or pattern discrimination; therefore, only S1–S4 were retained in the final model. Details of predictor selection are explained in S1 Text.

While lung mechanics exist on a continuous spectrum, a binary pattern classification (*k* = 2) was selected to maximize clinical utility. This choice was supported by statistical metrics (Elbow and Silhouette methods), clinical rationale (distinguishing obstructive from non-obstructive lung mechanics), and visual inspection of the TC sequences, which showed progressively increasing TCs in preliminary analyses (Fig 2). At *k* = 3, the additional cluster represented a ‘straight’ pattern of non-prolonged consecutive TCs (near-normal lung emptying) that did not capture obstructive pathology better than two clusters.

The model was then used to classify the entire cohort. For data visualization, Uniform Manifold Approximation and Projection (UMAP) was used to transform the predictors into two dimensions. Extensive details on model K-means clustering methodology and validation are provided in S2 Table*.*

### Statistical analysis

Continuous variables are reported as mean (SD) or median (IQR), and categorical variables as n (%). Overall spirometric differences between identified TC patterns were compared using Pearson’s Chi-squared test and the Welch Two-Sample *t* test, which accounts for unequal variances and sample sizes. Subsequent stratified comparisons between TC patterns within each spirometric category were performed using Wilcoxon Rank-Sum and Chi-squared tests, as determined by data distribution.

To further investigate differences in common spirometric variables between identified TC patterns, a stratified propensity score matching was performed to control for demographic confounders. Within each sex and spirometric category, patients presenting with the obstructive pattern group were matched 1:1 with patients from the normal pattern group using a nearest neighbor algorithm on a propensity score derived from age, weight, and height, with strict calipers applied to ensure balance. Following this matching, the balanced cohorts were compared using an independent *t* test. A *P*-value of <.05 was considered statistically significant. All statistical analyses and visualizations were performed with RStudio (R Foundation for Statistical Computing, Vienna, Austria, version 4.4.3).

## Results

The final cohort included 19,937 subjects with a mean (SD) age of 58 (15) years, of whom 45% were male. Baseline characteristics for each spirometric category are presented in Table 1.

**Table 1:** Baseline demographic and spirometric characteristics stratified by spirometric category.

Baseline characteristics				
**Characteristic**	**Normal**	**Possible restriction**	**Nonspecific FEV**_**1**_ **reduction**	**Obstruction**
	*n = 12,215*	*n = 3,233*	*n = 709*	*n = 3,780*
Age (yrs)	57 (15)	59 (15)	57 (16)	60 (15)
Sex Male (n, %)	5,158 (42%)	1,638 (51%)	323 (46%)	1,932 (51%)
Weight (kg)	83 (19)	89 (23)	86 (21)	79 (20)
Height (cm)	169 (10)	169 (10)	170 (10)	169 (10)
BMI (kg/m[2])	29 (6)	31 (7)	30 (6)	28 (6)
FEV_1_ (L)	2.9 (0.9)	1.9 (0.7)	2.2 (0.7)	1.8 (0.8)
FEV_1_ (z-score)	−0.24 (0.87)	−2.41 (0.81)	−1.86 (0.17)	−2.54 (1.15)
FVC (L)	3.7 (1.1)	2.4 (0.8)	3.1 (0.9)	3.0 (1.2)
FVC (z-score)	−0.09 (0.91)	−2.53 (0.89)	−1.34 (0.24)	−1.24 (1.46)
FEV_1_/FVC (%)	78 (6)	78 (8)	71 (4)	57 (9)
FEV_1_/FVC (z-score)	−0.11 (0.78)	−0.04 (1.07)	−1.09 (0.39)	−2.64 (0.82)
PEF (L/s)	6.8 (2.1)	5.0 (1.9)	5.4 (1.8)	4.3 (1.9)
PEF (z-score)	−0.45 (1.25)	−2.11 (1.35)	−1.81 (1.00)	−2.72 (1.37)
MEF_75_ (L/s)	5.9 (1.8)	4.4 (1.8)	4.2 (1.4)	2.6 (1.5)
MEF_75_ (z-score)	−0.29 (0.84)	−1.30 (0.92)	−1.43 (0.59)	−2.46 (0.77)
MEF_50_ (L/s)	3.3 (1.3)	2.4 (1.3)	2.0 (0.8)	1.2 (0.8)
MEF_50_ (z-score)	−0.65 (0.82)	−1.41 (0.84)	−1.78 (0.34)	−2.38 (0.45)
MEF_25_ (L/s)	1.1 (0.6)	0.7 (0.6)	0.6 (0.3)	0.4 (0.3)
MEF_25_ (z-score)	−0.02 (0.81)	−0.50 (0.98)	−0.92 (0.57)	−1.58 (0.83)
MMEF_25–75_ (L/s)	2.6 (1.1)	1.9 (1.1)	1.6 (0.7)	0.9 (0.7)
MMEF_25–75_ (z-score)	−0.34 (0.85)	−1.11 (0.99)	−1.59 (0.40)	−2.45 (0.72)
FEV_3_/FEV_6_ (%)	95 (3)	95 (3)	93 (3)	87 (5)
TC 63% (s)	0.56 (0.13)	0.56 (0.18)	0.74 (0.15)	1.38 (0.62)
S1 (s)	0.52 (0.38)	0.41 (0.32)	0.36 (0.24)	0.30 (0.16)
S2 (s)	0.40 (0.20)	0.34 (0.17)	0.39 (0.14)	0.67 (0.35)
S3 (s)	0.42 (0.15)	0.41 (0.17)	0.52 (0.15)	1.00 (0.52)
S4 (s)	0.58 (0.22)	0.63 (0.29)	0.82 (0.27)	1.56 (0.83)
S5 (s)	1.26 (0.97)	1.26 (0.85)	1.67 (0.98)	2.17 (1.37)

Data for continuous variables are presented as mean (standard deviation), while categorical variables are presented as n (%).BMI - Body Mass Index;TC 63% - Expiratory time constant at 63% of FVC; PEF - Peak Expiratory Flow; MEF - Maximal Expiratory Flow at specified percentage of forced vital capacity; MMEF - Maximal Mid-Expiratory Flow, FEV_3_/FEV_6_ – Forced Expiratory Volume at 3 and 6 seconds, S1 – S5: Expiratory time constants for volumetric slices 1–5; FEV_1_ - Forced expiratory volume in 1 second; FVC - Forced vital capacity.

### Identification and prevalence of time constant patterns

**Fig 3:**
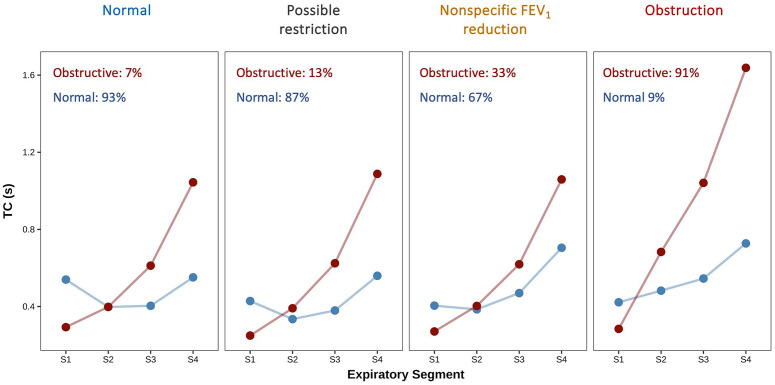
Time Constant Patterns Identified by Machine Learning Across Different Spirometric Categories. The obstructive pattern is characterized by a marked, progressive elevation of TC from early (S1) to late (S4) expiratory segments. In contrast, the normal pattern exhibits a significantly more homogenous and flatter pattern. Abbreviations: FEV1, Forced Expiratory Volume in 1 second; TC, Time Constant; S1–S4, compartmental expiratory time constants.

The K-means algorithm identified two distinct physiological phenotypes: a ‘normal pattern’ with stable TCs and an ‘obstructive pattern’ marked by progressively prolonging TCs (Fig 2). These patterns showed high agreement with standard classification: normal TC pattern appearing in 93% of normal subjects, and obstructive pattern was present in 91% of patients with established obstruction by LLN. An obstructive pattern was also identified in 7% of normal, 13% of possible restriction, and 33% of nonspecific FEV₁ reduction groups (Fig 3).

**Fig 4:**
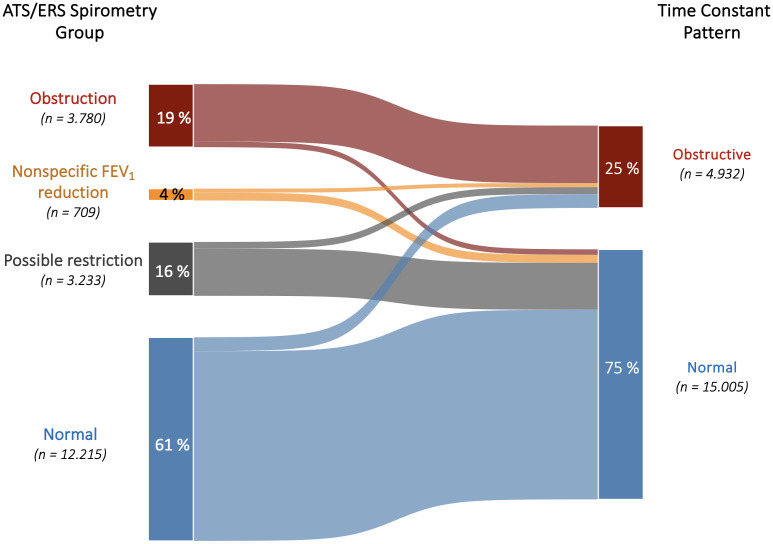
Transition Between Standard Spirometry Classification and K-means Derived TC Patterns. The Sankey diagram visualizes the reclassification of patients from their original spirometric categories by LLN to two pattern groups identified by the K-means clustering. Abbreviations: FEV1, Forced Expiratory Volume in 1 second; TC, Time Constant.

In total, an obstructive pattern was found in an additional 10% of subjects classified as non-obstructive by LLN. Transition between the standard spirometric classification and K-means derived patterns is shown in Fig 4.

**Fig 5:**
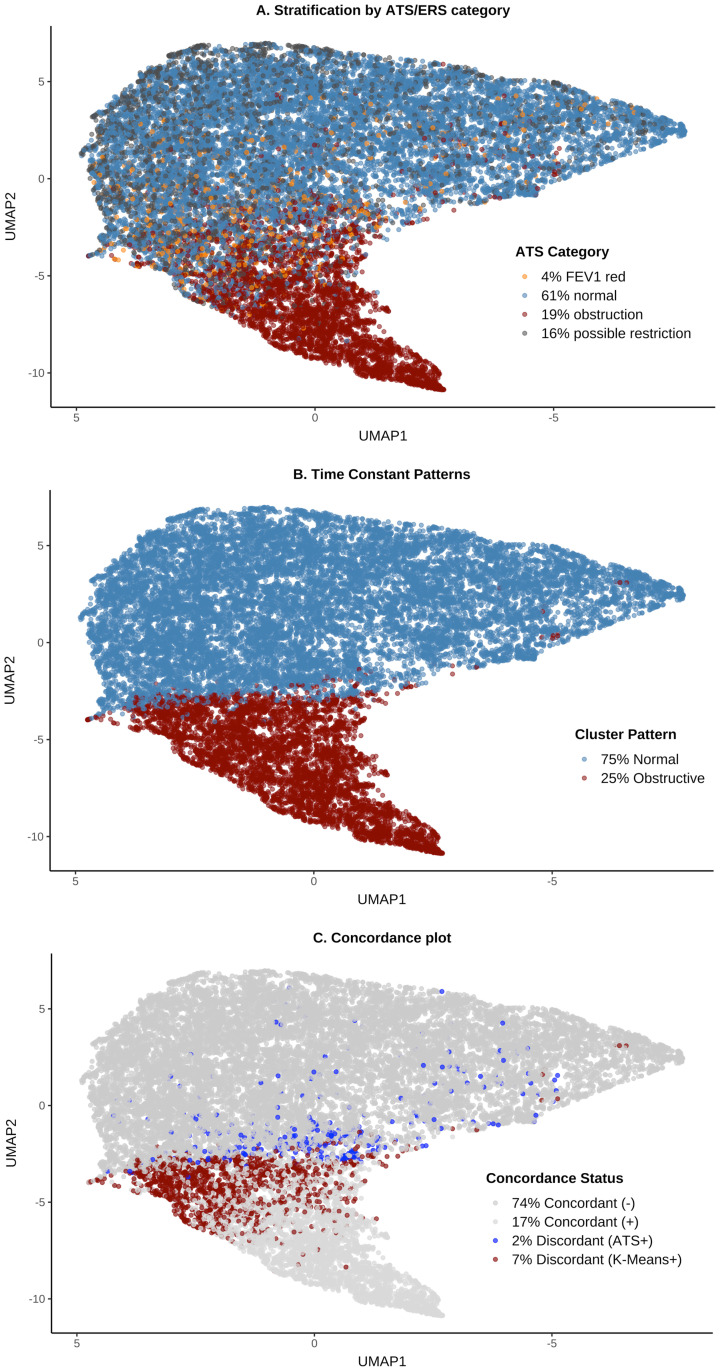
A Uniform Manifold Approximation and Projection (UMAP) of Spirometric Data. Each point represents an individual subject, with data colored according to three different classification schemes: (A) standard ATS/ERS spirometric categories; (B) machine learning-derived TC patterns (normal and obstructive); and (C) concordance plot, illustrating concordance between the ATS/ERS and K-means clustering classifications. Abbreviations: ATS/ERS, American Thoracic Society/European Respiratory Society; TC, expiratory time constant.

UMAP projections visualized these classifications, showing that while stratification by standard spirometric criteria resulted in largely overlapping groups (Fig 5A), the K-means-derived patterns produced two better separated clusters (Fig 5B). A concordance plot highlights the subjects classified with an obstructive pattern but not by traditional spirometric criteria (Fig 5C).

### Discordance analysis

The concordance plot revealed two discordant groups (Fig 5C): a ‘K-means–discordant’ group (7%), in which the TC pattern was obstructive despite non-obstructive spirometry by the LLN, and an ‘ATS-discordant’ group (2%), in which spirometry was obstructed by the LLN but the TC pattern was normal. The two groups had almost identical raw FEV₁/FVC ratios (67.4% vs. 67.5%; p = 0.82), yet their FEV₁/FVC z-scores diverged markedly (−2.03 vs. −1.20; p < 0.001). This divergence reflected a marked age difference: the ATS-discordant subjects were younger than the K-means–discordant subjects (mean age 39.6 vs. 70.3 years; p < 0.001). Despite their higher absolute spirometric values, their age-adjusted z-scores for FEV₁, FEV₁/FVC, and PEF were lower than those of the K-means–discordant group (Table 2).

**Table 2:** Comparison of spirometric characteristics between discordant groups.

Comparison of Spirometric Variables between Discordant groups			
	**ATS+ discordance** (n = 330)	**K-means+ Discordance** (n = 1,482)	***p***
Age (yrs)	39.6 (14.2)	70.3 (9.7)	<0.001
Sex Male (n, %)	125 (37.9%)	851 (57.4%)	<0.001
Weight (kg)	80.2 (19.0)	84.0 (19.5)	0.001
Height (cm)	171 (10.3)	169 (10.1)	<0.001
FEV_1_ (L)	2.6 (1.0)	2.0 (0.7)	<0.001
FEV_1_ (z-score)	−2.13 (1.44)	−1.50 (1.00)	<0.001
FVC (L)	3.7 (1.4)	3.0 (1.0)	<0.001
FVC (z-score)	−1.09 (1.91)	−0.92 (1.26)	0.14
FEV_1_/FVC (%)	67.4 (7.3)	67.5 (3.4)	0.82
FEV_1_/FVC (z-score)	−2.03 (0.57)	−1.20 (0.44)	<0.001
PEF (L/s)	5.6 (2.3)	5.1 (1.9)	0.002
PEF (z-score)	−2.22 (1.86)	−1.70 (1.26)	<0.001
MEF_75_ (L/s)	4.7 (1.6)	3.7 (1.4)	<0.001
MEF_75_ (z-score)	−1.43 (0.75)	−1.60 (0.69)	<0.001
MEF_50_ (L/s)	2.5 (0.9)	1.5 (0.6)	<0.001
MEF_50_ (z-score)	−1.79 (0.60)	−1.88 (0.36)	<0.01
MEF_25_ (L/s)	0.9 (0.5)	0.4 (0.2)	<0.001
MEF_25_ (z-score)	−1.29 (0.98)	−0.79 (0.62)	<0.001
MMEF_25–75_ (L/s)	2.1 (0.9)	1.1 (0.5)	<0.001
MMEF_25–75_ (z-score)	−1.92 (0.87)	−1.53 (0.51)	<0.001
FEV_3_/FEV_6_ (%)	92.4 (6.0)	90.9 (2.3)	<0.001
TC 63% (s)	0.73 (0.10)	0.87 (0.12)	<0.001

Comparisons were performed using independent t-tests for continuous variables and Chi-square test for categorical variables. **ATS+ discordance** represents a group with obstruction by LLN and a normal TC pattern, while **K-means+ Discordance** means obstruction defined by the presence of an obstructive TC pattern without obstruction by LLN. *Abbreviations:* TC 63% - Global expiratory time constant at 63% of FVC; PEF- Peak Expiratory Flow; MEF- Maximal Expiratory Flow at specified percentage of forced vital capacity; MMEF- Maximal Mid-Expiratory Flow; FEV_3_/FEV_6_– Ratio of Forced Expiratory Volume at 3 and 6 seconds; FEV_1_- Forced expiratory volume in 1 second; FVC- Forced vital capacity.

### Overall spirometric comparisons

Across all standard spirometric categories, subjects exhibiting the obstructive TC pattern demonstrated significantly poorer lung function compared to those with the normal TC pattern. This impairment was consistent across major domains, showing reduced ventilatory capacity (lower FEV₁ and FVC) and marked deficits in small airway function (lower MMEF_**25–75**_ and FEV_3_/FEV_6_). A detailed comparison between TC patterns and each spirometric group is provided in S2 Table.

### Propensity score matched comparisons

To eliminate demographic (especially age-related) differences in lung function, propensity score matching was performed, and confirmed that the obstructive pattern was universally associated with significantly poorer spirometry for each sex, after controlling for age, weight and height. This was evident across all spirometric categories, particularly in markers of small airway disease.

In the normal spirometry group, subjects with an obstructive pattern had a significantly lower FEV₁ (males: 2.7 vs. 3.0 L; females: 1.8 vs. 2.0 L; P < .001) and FEV₁/FVC (males: 67% vs. 76%; females: 68% vs. 78%; P < .001). This was accompanied by worse MMEF_25–75_ (males: 1.6 vs. 2.6 L/s; females: 1.0 vs. 1.8 L/s) and lower FEV_3_/FEV_6_ (males: 91% vs. 94%; females: 91% vs. 95%), with all comparisons being significant (P < .001). FVC was not different between the patterns and spirometric groups after matching (Table 3).

**Table 3:** Propensity Score Matching of TC Patterns for Males (3A) and Females (3B). Patients from the obstructive pattern group were matched 1:1 with patients from the normal pattern group using propensity scores based on age, weight, and height. Data are presented as mean (standard deviation), with p-values from an independent *t* test comparing the matched groups. FEV_1_ - forced expiratory volume in 1 second; FVC - forced vital capacity; PEF, peak expiratory flow; MEF_75_, MEF_50_, MEF_25_ - maximal expiratory flow at 75%, 50%, and 25% of FVC, respectively; MMEF_25−75_ - maximal mid-expiratory flow; FEV_3_/FEV_6_ - ratio of forced expiratory volume in 3 and 6 seconds; TC 63%, expiratory time constant (measured time to exhale first 63% of FVC).

A. Comparison of Spirometric Variables between patterns after Propensity Score Matching in Males.												
Group	Normal			Possible restriction			Nonspecific FEV_1_ reduction			Obstruction		
Pattern	Normal	Obstructive	*p*	Normal	Obstructive	*p*	Normal	Obstructive	*p*	Normal	Obstructive	*p*
*n*	*442*	*442*		*242*	*242*		*41*	*41*		*105*	*105*	
**Age** (yrs)	69 (10)	69 (10)	0.98	69 (9)	69 (9)	0.9	62 (10)	62 (11)	0.96	42 (15)	43 (15)	0.66
**Weight** (kg)	88 (14)	88 (15)	0.98	95 (18)	95 (18)	0.98	93 (14)	92 (15)	0.78	87 (13)	87 (13)	0.88
**Height** (cm)	175 (7)	176 (7)	0.63	174 (7)	174 (7)	0.91	176 (6)	177 (6)	0.44	179 (7)	178 (7)	0.14
**FEV**_**1**_ (L)	3.0 (0.7)	2.7 (0.6)	**<0.001**	2 (0.5)	1.7 (0.4)	**<0.001**	2.5 (0.5)	2.4 (0.4)	0.67	3.0 (1)	2.7 (1)	**0.04**
**FVC** (L)	4 (0.8)	4 (0.8)	0.19	2.5 (0.6)	2.5 (0.6)	0.45	3.5 (0.7)	3.6 (0.6)	0.4	4.4 (1.5)	4.4 (1.5)	0.92
**FEV**_**1**_**/FVC** (%)	76 (5)	67 (3)	**<0.001**	77 (6)	68 (5)	**<0.001**	71 (3)	67 (3)	**<0.001**	66 (9)	59 (8)	**<0.001**
**PEF** (L/s)	7.6 (1.9)	6.8 (1.6)	**<0.001**	5.5 (1.8)	4.5 (1.4)	**<0.001**	6.2 (1.5)	6.2 (1.1)	0.98	6.6 (2.8)	6.1 (2.4)	0.21
**MEF**_**75**_ (L/s)	6.5 (1.7)	4.9 (1.2)	**<0.001**	4.8 (1.7)	3.2 (1)	**<0.001**	4.9 (1.07)	4.3 (0.91)	**0.01**	5.6 (1.6)	3.6 (1.8)	**<0.001**
**MEF**_**50**_ (L/s)	3.4 (1.1)	2 (0.59)	**<0.001**	2.5 (1.1)	1.3 (0.4)	**<0.001**	2.3 (0.6)	1.8 (0.4)	**<0.001**	3 (1)	1.8 (1)	**<0.001**
**MEF**_**25**_ (L/s)	0.97 (0.4)	0.56 (0.2)	**<0.001**	0.67 (0.4)	0.35 (0.1)	**<0.001**	0.7 (0.30)	0.53 (0.20)	**0.001**	1 (0.5)	0.7 (0.4)	**<0.001**
**MMEF**_**25–75**_ (L/s)	2.6 (0.9)	1.6 (0.5)	**<0.001**	1.8 (0.8)	0.9 (0.3)	**<0.001**	1.7 (0.45)	1.4 (0.4)	**<0.001**	2.4 (0.9)	1.5 (0.8)	**<0.001**
**FEV**_**3**_**/FEV**_**6**_ (%)	94 (2)	91 (2)	**<0.001**	95 (2)	91 (3)	**<0.001**	92 (2)	91 (2)	**<0.001**	92 (8)	88 (5)	**<0.001**
**TC 63%** (s)	0.57 (0.11)	0.86 (0.1)	**<0.001**	0.53 (0.14)	0.89 (0.14)	**<0.001**	0.71 (0.1)	0.87 (0.1)	**<0.001**	0.75 (0.1)	1.27 (0.54)	**<0.001**
B. Comparison of Spirometric Variables between patterns after Propensity Score Matching in Females.												
Group	Normal			Possible restriction			Nonspecific FEV_1_ reduction			Obstruction		
Pattern	Normal	Obstructive	*p*	Normal	Obstructive	*p*	Normal	Obstructive	*p*	Normal	Obstructive	*p*
	*n = 365*	*n = 365*		*n = 136*	*n = 136*		*n = 53*	*n = 53*		*n = 152*	*n = 152*	
**Age** (yrs)	72 (9.8)	72 (9.7)	0.95	72 (8)	72 (9)	0.85	69 (7.7)	70 (7.8)	0.78	43 (14)	44 (14)	0.59
**Weight** (kg)	73 (15)	73 (15)	0.87	77 (16)	77 (16)	0.99	77 (13)	77 (14)	0.94	72 (13)	71 (13)	0.6
**Height** (cm)	161 (6)	161 (7)	0.61	160 (6)	160 (6)	0.99	162 (6)	162 (7)	0.88	166 (7)	165 (6)	0.47
**FEV**_**1**_ (L)	2.0 (0.5)	1.8 (0.4)	**<0.001**	1.3 (0.4)	1.1 (0.3)	**0.001**	1.6 (0.3)	1.5 (0.3)	0.2	2.2 (0.8)	2.0 (0.6)	**0.01**
**FVC** (L)	2.6 (0.6)	2.7 (0.6)	0.27	1.6 (0.4)	1.7 (0.4)	0.25	2.2 (0.4)	2.2 (0.4)	0.69	3.2 (1.1)	3.2 (0.8)	0.67
**FEV**_**1**_**/FVC** (%)	78 (5)	68 (3)	**<0.001**	78 (7)	68 (3)	**<0.001**	72 (3)	67 (2)	**<0.001**	68 (6)	62 (6)	**<0.001**
**PEF** (L/s)	5 (1.1)	4.5 (1.1)	**<0.001**	3.6 (1.1)	3 (0.90)	**<0.001**	4.1 (1)	3.9 (1)	0.31	4.9 (1.8)	4.6 (1.3)	0.11
**MEF**_**75**_ (L/s)	4.5 (1.1)	3.4 (0.9)	**<0.001**	3.2 (1.1)	2.2 (0.8)	**<0.001**	3.5 (0.9)	2.8 (0.9)	**<0.001**	4 (1.2)	2.9 (1)	**<0.001**
**MEF**_**50**_ (L/s)	2.5 (0.9)	1.4 (0.4)	**<0.001**	1.7 (0.8)	0.83 (0.3)	**<0.001**	1.54 (0.5)	1.14 (0.3)	**<0.001**	2.2 (0.8)	1.4 (0.6)	**<0.001**
**MEF**_**25**_ (L/s)	0.66 (0.3)	0.36 (0.2)	**<0.001**	0.5 (0.4)	0.24 (0.1)	**<0.001**	0.4 (0.16)	0.3 (0.1)	**<0.001**	0.75 (0.4)	0.5 (0.2)	**<0.001**
**MMEF**_**25–75**_ (L/s)	1.8 (0.7)	1 (0.3)	**<0.001**	1.3 (0.7)	0.6 (0.2)	**<0.001**	1.1 (0.4)	0.8 (0.2)	**<0.001**	1.7 (0.7)	1.1 (0.5)	**<0.001**
**FEV**_**3**_**/FEV**_**6**_ (%)	95 (2)	91 (2)	**<0.001**	95 (3)	92 (3)	**<0.001**	92 (4)	91 (1)	**0.01**	92 (5)	89 (4)	**<0.001**
**TC 63%** (s)	0.55 (0.12)	0.83 (0.1)	**<0.001**	0.52 (0.15)	0.87 (0.1)	**<0.001**	0.66 (0.1)	0.85 (0.1)	**<0.001**	0.73 (0.1)	1.11 (0.4)	**<0.001**

This distinction was also clear in the nonspecific FEV_1_ reduction group, where, despite a non-significant difference in FEV_1_, the FEV_1_/FVC (males: 67% vs. 71%; females: 67% vs. 72%; P < .001) and MMEF_25–75_ (males: 1.4 vs. 1.7 L/s; females: 0.8 vs. 1.1 L/s; P < .001) remained worse for those having an obstructive pattern. A similar trend was also observed for the possible restriction category (Table 3).

### Isolated small-airway dysfunction

To determine whether the TC approach captures small-airway dysfunction not reflected in the FEV_1_/FVC ratio, we examined subjects non-obstructed by the LLN (FEV_1_/FVC z ≥ −1.645) and defined isolated small-airway dysfunction as an MMEF_25–75_ below the LLN (<−1.645) [8]. The obstructive TC pattern was markedly more prevalent in subjects with isolated small-airway dysfunction than in those with preserved MMEF_25–75_ (33.5% vs. 6.0%; P < .001), representing more than a fivefold increase. A full comparison between the two groups is shown in S3 Table.

### Obstruction group by LLN

Even within the formally classified obstruction group (FEV_1_/FVC < LLN), individuals identified with the normal pattern were younger (mean age 43 years for both sexes) and had significantly better lung mechanics, showing a higher FEV_1_ (males: 3.0 vs. 2.7 L, P = .04; females: 2.2 vs. 2.0 L, P = .01) and FEV_1_/FVC (males: 66% vs. 59%; females: 68% vs. 62%; P < .001 for both) than those with the obstructive pattern. Similarly, MMEF_25–75_ (males: 2.4 vs.1.5 L/s; females: 1.7 vs. 1.1 L/s) and FEV_3_/FEV_6_ (males: 92%vs. 88%; females: 92% vs. 89%) were also significantly higher (P < .001 for all) in favor of the normal pattern (Table 3).

## Discussion

This study identified an obstructive mechanics of lung emptying, characterized by a consecutive prolongation of compartmental TCs, in an additional 10% of individuals classified as non-obstructive by current criteria. Patients with this spirometric physiologic phenotype displayed significantly worse spirometric parameters, suggesting that TC pattern analysis can uncover altered lung emptying mechanics not evident by classification based on LLN.

The TC is fundamentally determined by the product of Raw and Crs, meaning an increase in either variable will prolong the TC. This research is founded on that principle, specifically focusing on Raw, as an increase in Raw is the predominant physiological hallmark of obstructive lung mechanics [17]. While traditionally assessed in passive mechanical ventilation, the TC’s application extends to forced spirometric maneuvers [23]. As the product of Raw and Crs, the TC is a governing parameter of lung emptying; although the absolute volumes and flows differ between passive and forced maneuvers, the ratio of volume to flow remains constant [26]. Previous research has highlighted the utility of using a single, global TC to identify obstruction. Ikeda et al. proposed a TC cutoff of 0.6s calculated from mid-expiratory flow (MEF_50−25_) [27], while Bodduluri et al. established a reference upper limit of normal for measured TC in healthy individuals in the NHANES III dataset to be 0.76s.^19^ Notably, applying this cutoff to the COPDGene cohort identified airflow obstruction in an additional 20% of smokers missed by the gold standard [19].

While these single TC values are informative, the concept can be improved further. The slicing method, adapted from Guttmann, analyzes a sequence of compartmental TCs that reflect changes throughout forced exhalation [20]. This provides a more complete representation of expiratory mechanics than a single global TC. Unlike traditional metrics that capture volumes (FEV₁, FVC) and flows (MEFs) at isolated time points, TC analysis assesses the direct relationship between volume and flow across the entire maneuver (Fig 2). While reduced flows inherently prolong global TCs, the slicing method offers a more granular approach: while FEV_1_/FVC or FEV_1_ < LLN indicate possible obstruction, the TC sequence shows a more complete picture of where and how it evolves throughout exhalation, distinguishing mechanical properties from simple overall flow limitation.

A persistent challenge in spirometry is differentiating a true restrictive pathology from combined obstructive/restrictive disease, as both can present with a reduced FVC [28,29]. Although the definitive diagnosis of restriction requires documentation of reduced total lung capacity, which is beyond the capability of spirometry, TC patterns may help resolve this ambiguity. Among the cohort classified as possible restriction based on FVC < LLN, a significant proportion (13%) exhibited an obstructive pattern, suggesting a combined process rather than a primary restrictive pathology. In both sexes, values for FEV_1_ and FEV₁/FVC were significantly different between the normal and obstructive patterns, while values for FVC were not. TC pattern analysis may therefore serve as an additional tool to unmask a probable obstructive component in patients with a seemingly restrictive spirometric pattern by the current classification.

A notable finding was a subgroup of younger patients (mean age of 43 years) with confirmed spirometric obstruction by LLN who paradoxically presented with a normal TC pattern. Although meeting the FEV_1_/FVC threshold for obstruction by LLN, this cohort exhibited significantly higher FEV_1_ and FEV_1_/FVC, while FVC was preserved (4.4 L in males and 3.2L in females). In these patients, small airway disease metrics, including MMEF_25–75_ and FEV_3_/FEV_6,_ were also significantly better. These findings align with the concept of dysanapsis, where airflow limitation with a preserved FVC is a consequence of a structural state rather than acquired small airway disease [30,31]. Slicing method may therefore help distinguish physiological airway disease from dysanaptic lung growth, a concept requiring further research.

Mechanistically, a structurally smaller but otherwise healthy lung should empty homogeneously, yielding non-increasing consecutive TCs, whereas acquired small-airway disease introduces a rather heterogeneous lung parenchyma, manifesting as progressively delayed emptying (a rising consecutive TCs). This distinction may explain why individuals with dysanapsis, despite a reduced FEV_1_/FVC, may retain a near-normal TC pattern. Moreover, these were younger individuals (mean age 43 years), entering the age window of risk for COPD, further supporting a structural (dysanaptic) rather than an acquired, disease-driven origin of their airflow limitation.

Both electrical impedance tomography (EIT) and spirometric ‘slicing’ assess expiratory TCs, but they provide different perspectives on lung emptying. EIT creates anatomical maps that visualize the spatial distribution of fast and slow-emptying lung units, revealing regional ventilation heterogeneity [32]. In contrast, the slicing method provides a functional map, ordering lung units by their emptying sequence—from the fastest (S1) to progressively slower units (S2-S5)—irrespective of their anatomical location. Qu et al. proposed averaging regional EIT data to derive a single global TC, particularly as heterogeneity can be significant in diseases like COPD. This approach, however, reduces the complex dynamics of exhalation to a single value [33].

This study has several limitations. Its retrospective design precludes conclusions about causality or longitudinal outcomes. The normal subgroup used to train the clustering model was drawn from a clinical population with objectively normal spirometry rather than a healthy general population cohort; although pragmatic, such individuals may harbor respiratory symptoms or subclinical disease, and a strictly healthy reference would refine the normal pattern and is warranted for future validation. The absence of specific clinical diagnoses prevented mapping the identified physiological phenotypes onto distinct disease states, leaving it unclear whether the obstructive pattern is disease-specific or simply reflects increased Raw (or associated changes in Crs) of any cause. Finally, the terminal compartment (S5) yielded negative, non-representative values in 8% of subjects — a technical artifact of the low airflows at end-exhalation, where terminal effort and measurement noise dominate; this affected neither pattern recognition nor the final model.

## Conclusions

Our study introduces compartmental TC analysis to standardize the interpretation of the flow-volume curve. Because this technique relies on standard flow/volume data, it can be seamlessly integrated into routine spirometers or body plethysmographs. This granular view may offer a diagnostic advantage, particularly in ambiguous cases like ‘possible restriction’ and in mixed obstructive/restrictive ventilatory defects, where TC analysis can unmask a probable underlying obstructive component confounded by volume reduction, potentially refining the differential diagnosis. Future work should focus on validating these findings by associating TC patterns with specific disease states and exploring their relationships with imaging and clinical outcomes.

## Supporting information

S1 Table Summary of compartmental volumes, expiratory times, and calculated expiratory time constants. (DOCX)

S2 Table Detailed comparison between time-constant patterns and each spirometric group. (DOCX)

S3 Table Spirometric characteristics of subjects with versus without isolated small-airway dysfunction among those with a preserved FEV₁/FVC ratio. (DOCX)

S1 Text Predictor selection, K-means clustering methodology, and model validation. (DOCX)

S1 Data De-identified dataset underlying the study findings. (CSV)

## References

[1] Venkatesan P. GOLD COPD report: 2025 update. Lancet Respir Med. 2025;13:e7–8. 10.1016/S2213-2600(24)00413-2 39647487

[2] Adeloye D, Song P, Zhu Y, Campbell H, Sheikh A, Rudan I, et al. Global, regional, and national prevalence of, and risk factors for, chronic obstructive pulmonary disease (COPD) in 2019: A systematic review and modelling analysis. Lancet Respir Med. 2022;10(5):447–58. doi: 10.1016/S2213-2600(21)00511-7 35279265 PMC9050565

[3] European Respiratory Society. European Lung White Book. Sheffield, UK: European Respiratory Society; 2013 [cited 2025 Oct 8]. https://www.erswhitebook.org/

[4] Hansen JE, Sun X-G, Wasserman K. Spirometric criteria for airway obstruction: Use percentage of FEV1/FVC ratio below the fifth percentile, not < 70%. Chest. 2007;131(2):349–55. doi: 10.1378/chest.06-1349 17296632

[5] Miller MR, Hankinson J, Brusasco V, Burgos F, Casaburi R, Coates A, et al. Standardisation of spirometry. Eur Respir J. 2005;26(2):319–38. doi: 10.1183/09031936.05.00034805 16055882

[6] Quanjer PH, Stanojevic S, Cole TJ, Baur X, Hall GL, Culver BH, et al. Multi-ethnic reference values for spirometry for the 3-95-yr age range: The global lung function 2012 equations. Eur Respir J. 2012;40(6):1324–43. doi: 10.1183/09031936.00080312 22743675 PMC3786581

[7] Barjaktarevic IZ, Cooper CB. The role of small airways in the development of chronic obstructive pulmonary disease: A state-of-the-art review. Chest. 2022;162:801–13.

[8] Knox-Brown B, Potts J, Santofimio VQ, Minelli C, Patel J, Abass NM, et al. Isolated small airways obstruction predicts future chronic airflow obstruction: A multinational longitudinal study. BMJ Open Respir Res. 2023;10(1):e002056. doi: 10.1136/bmjresp-2023-002056 37989490 PMC10660204

[9] McFadden ER, Linden DA. A reduction in maximum mid-expiratory flow rate. A spirographic manifestation of small airway disease. Am J Med. 1972;52(6):725–37. doi: 10.1016/0002-9343(72)90078-2 5030170

[10] Graham BL, Steenbruggen I, Miller MR, Barjaktarevic IZ, Cooper BG, Hall GL, et al. Standardization of spirometry 2019 update. An Official American Thoracic Society and European Respiratory Society Technical Statement. Am J Respir Crit Care Med 2019;200:e70–e88. 10.1164/rccm.201908-1590ST PMC6794117 31613151

[11] Ferguson GT, Enright PL, Buist AS, Higgins MW. Office spirometry for lung health assessment in adults: A consensus statement from the National Lung Health Education Program. Chest. 2000;117(4):1146–61. doi: 10.1378/chest.117.4.1146 10767253

[12] Yee N, Markovic D, Buhr RG, Fortis S, Arjomandi M, Couper D. Significance of FEV3/FEV6 in recognition of early airway disease in smokers at risk of development of COPD: Analysis of the SPIROMICS Cohort. Chest. 2022;161:949–59. 34767825 10.1016/j.chest.2021.10.046 PMC9005864

[13] Morris ZQ, Coz A, Starosta D. An isolated reduction of the FEV3/FVC ratio is an indicator of mild lung injury. Chest. 2013;144(4):1117–23. doi: 10.1378/chest.12-2816 23493987

[14] Lam AHS, Alhajri SA, Potts J, Harrabi I, Anand MP, Janson C, et al. Optimal spirometry thresholds for the prediction of chronic airflow obstruction: A multinational longitudinal study. ERJ Open Res. 2025;11(2):00624–2024. doi: 10.1183/23120541.00624-2024 40040898 PMC11873882

[15] Dransfield MT, Kunisaki KM, Strand MJ, Anzueto A, Celli BR, Criner GJ. The shape of the expiratory flow-volume curve in smokers with and without COPD. Am J Respir Crit Care Med. 2008;177:355–60.

[16] Han MK, Kazerooni EA, Lynch DA, Liu LX, Murray S, Curtis JL, et al. Chronic obstructive pulmonary disease exacerbations in the COPDGene study: Associated radiologic phenotypes. Radiology. 2011;261(1):274–82. doi: 10.1148/radiol.11110173 21788524 PMC3184233

[17] Depta F, Chiofolo CM, Chbat NW, Euliano NR, Gentile MA, Rybár D, et al. Six methods to determine expiratory time constants in mechanically ventilated patients: A prospective observational physiology study. Intensive Care Med Exp. 2024;12(1):25. doi: 10.1186/s40635-024-00612-z 38451334 PMC10920606

[18] Depta F, Kallet RH, Gentile MA, Kassis ENB. Expiratory time constants in mechanically ventilated patients: Rethinking the old concept-a narrative review. Intensive Care Med Exp. 2025;13(1):40. doi: 10.1186/s40635-025-00745-9 40140183 PMC11947344

[19] Bodduluri S, Nakhmani A, Bhatt SP. Spirometric time constant for detection of early and mild airflow obstruction. Am J Respir Crit Care Med. 2024;209:A4680. 10.1164/rccm.202312-2301LE PMC11531222 38301239

[20] Guttmann J, Eberhard L, Fabry B, Bertschmann W, Zeravik J, Adolph M, et al. Time constant/volume relationship of passive expiration in mechanically ventilated ARDS patients. Eur Respir J. 1995;8(1):114–20. doi: 10.1183/09031936.95.08010114 7744177

[21] Lourens MS, van den Berg B, Aerts JG, Verbraak AF, Hoogsteden HC, Bogaard JM. Expiratory time constants in mechanically ventilated patients with and without COPD. Intensive Care Med. 2000;26(11):1612–8. doi: 10.1007/s001340000632 11193266

[22] Stanojevic S, Kaminsky DA, Miller MR, Thompson B, Aliverti A, Barjaktarevic I, et al. ERS/ATS technical standard on interpretive strategies for routine lung function tests. Eur Respir J. 2022;60(1):2101499. doi: 10.1183/13993003.01499-2021 34949706

[23] Depta F, Bodnárová S, Šurinová K, Paraničová I, Sokolová E, Lombartová E, et al. Expiratory time constants in patients undergoing routine spirometry. CHEST Pulmonary. 2025;3(3):100181. doi: 10.1016/j.chpulm.2025.100181 42548344 PMC13418014

[24] Depta F, Hoheisel A, Bhatt SP, Koblížek V, Bodduluri S, Stolz D. Four methods to determine expiratory time constants from spirometry and their sensitivity to detect airway obstruction. Lung. 2026;204:3. 41484401 10.1007/s00408-025-00866-8

[25] Haraty RA, Dimishkieh M, Masud M. An enhanced k-means clustering algorithm for pattern discovery in healthcare data. International Journal of Distributed Sensor Networks. 2015;11(6):615740. doi: 10.1155/2015/615740

[26] Pierce JA. Studies of free collapse in the intact human lung. J Lab Clin Med. 1959;54(1):96–106. 13665155

[27] Ikeda T, Yamauchi Y, Uchida K, Oba K, Nagase T, Yamada Y. Reference value for expiratory time constant calculated from the maximal expiratory flow-volume curve. BMC Pulm Med. 2019;19(1):208. doi: 10.1186/s12890-019-0976-6 31711456 PMC6849182

[28] Leuppi JD, Miedinger D, Chhajed PN, Buess S, Schafroth S, Tamm M. A reduced FEV1/FVC ratio in combination with a low FVC is a common finding in patients with a restrictive spirometric pattern. Respir Med. 2006;100.

[29] Balfe DL, Lewis M, Mohsenifar Z. The mixed ventilatory defect: Is it a distinct clinical entity?. Respir Care. 2015;60:1589–94.

[30] Lange P, Celli B, Agustí A, Boje Jensen G, Divo M, Faner R, et al. Lung-function trajectories leading to chronic obstructive pulmonary disease. N Engl J Med. 2015;373(2):111–22. doi: 10.1056/NEJMoa1411532 26154786

[31] Smith BM, Kirby M, Hoffman EA, Kronmal RA, Aaron SD, Allen NB, et al. Association of dysanapsis with chronic obstructive pulmonary disease among older adults. JAMA. 2020;323(22):2268–80. doi: 10.1001/jama.2020.6918 32515814 PMC7284296

[32] Waldmann AD, Czepluch AM, Schullcke B. Standalone electrical impedance tomography predicts spirometry indicators and enables regional lung assessment. Scientific Reports. 2022;12:16441. doi: 10.1038/s41598-022-20845-5 36085816

[33] Qu S, Feng E, Dong D, Yang L, Dai M, Frerichs I, et al. Early screening of lung function by electrical impedance tomography in people with normal spirometry reveals unrecognized pathological features. Nat Commun. 2025;16(1):622. doi: 10.1038/s41467-024-55505-2 39805822 PMC11731049

