# Supplementary material for: Expiratory time constant patterns reveal obstructive lung mechanics in non-obstructive spirometry
Source: PLOS Digit Health. 2026 Aug 11;5(8):e0001653. doi: 10.1371/journal.pdig.0001653 (PMC13460623; doi:10.1371/journal.pdig.0001653)
Supplement: Supplementary file 1 [file pdig.0001653.s001.docx]

**S1 Table**

**Supplemental Table 1: Descriptive statistics of compartmental TC and associated metrics stratified by spirometric category.** The expiratory portion of the flow-volume curve, from PEF to the end of exhalation, was divided into five compartments of equal volume. Compartmental expiratory time constants (S1-S5) were calculated for each compartment as the compartmental volume and its corresponding slope of flow. Data are presented as median (IQR). *Abbreviations:* S1-S5 (s) - Expiratory time constants for each volumetric compartment; Time S1-S5 (s) - Measured duration of each volumetric compartment; S1-S5 volume (L) - Volume of each of the five respective compartments. Compartmental TC boxplots for each spirometric category are shown in Figure 2.

| **Supplemental Table1** | | | | |
| --- | --- | --- | --- | --- |
| **Characteristic** | **Normal** | **Possible restriction** | **Nonspecific FEV_1_ reduction** | **Obstruction** |
|  | N = 12,215 | N = 3,233 | N = 709 | N = 3,780 |
| **S1** (s) | 0.41 (0.26, 0.66) | 0.30 (0.19, 0.51) | 0.28 (0.20, 0.44) | 0.25 (0.19, 0.35) |
| **S2** (s) | 0.35 (0.26, 0.47) | 0.30 (0.23, 0.40) | 0.37 (0.29, 0.47) | 0.57 (0.44, 0.78) |
| **S3** (s) | 0.39 (0.31, 0.50) | 0.39 (0.29, 0.50) | 0.50 (0.42, 0.60) | 0.84 (0.66, 1.17) |
| **S4** (s) | 0.55 (0.43, 0.70) | 0.58 (0.42, 0.79) | 0.80 (0.64, 0.97) | 1.35 (1.00, 1.89) |
| **S5** (s) | 1.2 (0.7, 1.7) | 1.2 (0.7, 1.7) | 1.6 (1.0, 2.2) | 2.1 (1.2, 3.0) |
| **Time S1** (s) | 0.10 (0.09, 0.11) | 0.09 (0.07, 0.11) | 0.12 (0.10, 0.13) | 0.18 (0.14, 0.22) |
| **Time S2** (s) | 0.15 (0.13, 0.18) | 0.15 (0.12, 0.19) | 0.20 (0.18, 0.23) | 0.33 (0.27, 0.45) |
| **Time S3** (s) | 0.26 (0.22, 0.33) | 0.28 (0.21, 0.38) | 0.38 (0.32, 0.46) | 0.66 (0.50, 0.89) |
| **Time S4** (s) | 0.7 (0.5, 0.9) | 0.76 (0.5, 1.0) | 1.0 (0.8, 1.3) | 1.6 (1.2, 2.0) |
| **Time S5** (s) | 6.1 (4.7, 8.2) | 5.80 (4.3, 7.4) | 6.2 (4.8, 8.6) | 5.7 (4.2, 7.9) |
| **S1 volume** (L) | 0.57 (0.46, 0.72) | 0.38 (0.29, 0.48) | 0.51 (0.39, 0.62) | 0.51 (0.38, 0.66) |
| **S2 volume** (L) | 0.60 (0.48, 0.75) | 0.40 (0.30, 0.51) | 0.53 (0.41, 0.65) | 0.52 (0.40, 0.68) |
| **S3 volume** (L) | 0.60 (0.48, 0.75) | 0.40 (0.31, 0.50) | 0.53 (0.41, 0.65) | 0.52 (0.40, 0.68) |
| **S4 volume** (L) | 0.60 (0.48, 0.75) | 0.40 (0.30, 0.50) | 0.53 (0.41, 0.65) | 0.52 (0.40, 0.68) |
| **S5 volume** (L) | 0.53 (0.37, 0.69) | 0.38 (0.26, 0.49) | 0.48 (0.35, 0.61) | 0.47 (0.31, 0.63) |
