# Supplementary material for: Expiratory time constant patterns reveal obstructive lung mechanics in non-obstructive spirometry
Source: PLOS Digit Health. 2026 Aug 11;5(8):e0001653. doi: 10.1371/journal.pdig.0001653 (PMC13460623; doi:10.1371/journal.pdig.0001653)
Supplement: Supplementary file 2 [file pdig.0001653.s002.docx]

**S2 Table**

**Table 3. Comparison of demographic and spirometric characteristics between TC patterns, stratified by spirometric category, before propensity score matching.** *P*-values were derived from the Wilcoxon Rank-Sum test for continuous variables and the Chi-squared test for categorical variables to compare the normal and obstructive pattern groups. Continuous variables are presented as mean (SD), and categorical variables are presented as n (%). *Abbreviations:* FEV_1_ - forced expiratory volume in 1 second; FVC - forced vital capacity; PEF, peak expiratory flow; MEF_75_, MEF_50_, MEF_25_ - maximal expiratory flow at 75%, 50%, and 25% of FVC, respectively; MMEF_25−75_ - maximal mid-expiratory flow; FEV_3_/FEV_6_ - ratio of forced expiratory volume in 3 and 6 seconds; TC 63%, expiratory time constant (measured time to exhale first 63% of FVC)

| **Demographic and Spirometric Characteristics between TC Patterns Across Spirometric Groups** | | | | | | | | | | | | |
| --- | --- | --- | --- | --- | --- | --- | --- | --- | --- | --- | --- | --- |
|  | **Normal** | | | **Possible restriction** | | | **Nonspecific**  **FEV_1_ reduction** | | | **Obstruction** | | |
| **Pattern** | **Normal** | **Obstructive** | ***P*** | **Normal** | **Obstructive** | ***P*** | **Normal** | **Obstructive** | ***P*** | **Normal** | **Obstructive** | ***P*** |
| **N**  (%) | 11.385  (93) | 830  (7) |  | 2.818  (87) | 415  (13) |  | 472  (67) | 237  (33) |  | 330  (9) | 3.450  (91) |  |
| **Age**  (yrs) | 57  (23) | 72  (12) | **<0.001** | 59  (22) | 72  (11) | **<0.001** | 51  (24) | 71  (12) | **<0.001** | 38  (18) | 65  (18) | **<0.001** |
| **Sex**  (Male) | 4699 (41%) | 459  (55%) | **<0.001** | 1373 (49%) | 265  (64%) | **<0.001** | 196 (42%) | 127  (54%) | **0.003** | 125 (38%) | 1807  (52%) | **<0.001** |
| **Weight**  (kg) | 82  (25) | 81  (25) | **0.02** | 86  (30) | 87  (28) | 0.87 | 85  (28) | 84  (28) | 0.65 | 78  (25) | 77  (25) | 0.2 |
| **Height**  (cm) | 168 (14) | 169  (14) | 0.36 | 169 (15) | 169  (15) | 0.93 | 170 (14) | 170  (17) | 0.33 | 170 (13) | 168  (14) | **<0.001** |
| **FEV_1_**  (L) | 2.8 (1.2) | 2.2  (0.9) | **<0.001** | 1.9 (0.9) | 1.4  (0.6) | **<0.001** | 2.4 (1.0) | 1.8  (0.9) | **<0.001** | 2.6 (1.2) | 1.6  (1) | **<0.001** |
| **FVC**  (L) | 3.6 (1.5) | 3.3  (1.4) | **<0.001** | 2.4 (1.2) | 2.1  (0.9) | **<0.001** | 3.2 (1.3) | 2.8  (1.4) | **<0.001** | 3.8 (1.5) | 2.8  (1.5) | **<0.001** |
| **FEV_1_/FVC**  (%) | 79  (7) | 68  (3) | **<0.001** | 79  (9) | 67  (4) | **<0.001** | 73  (5) | 67  (3) | **<0.001** | 70  (4) | 58  (12) | **<0.001** |
| **PEF**  (L/s) | 6.5 (2.9) | 5.6  (2.6) | **<0.001** | 4.9 (2.5) | 3.7  (2) | **<0.001** | 5.4 (2.2) | 4.7  (2.3) | **<0.001** | 5.6 (3.0) | 3.9  (2.4) | **<0.001** |
| **MEF_75_**  (L/s) | 5.8 (2.4) | 4.1  (1.8) | **<0.001** | 4.4 (2.3) | 2.7  (1.3) | **<0.001** | 4.6 (1.7) | 3.2  (1.7) | **<0.001** | 4.4 (1.9) | 2.1  (1.8) | **<0.001** |
| **MEF_50_**  (L/s) | 3.3 (1.6) | 1.7  (0.7) | **<0.001** | 2.3 (1.2) | 1.1  (0.5) | **<0.001** | 2.3 (1.1) | 1.3  (0.6) | **<0.001** | 2.4 (0.9) | 0.9  (0.8) | **<0.001** |
| **MEF_25_**  (L/s) | 1.0 (0.7) | 0.4  (0.2) | **<0.001** | 0.7 (0.6) | 0.3  (0.2) | **<0.001** | 0.7 (0.5) | 0.3  (0.2) | **<0.001** | 0.8 (0.5) | 0.3  (0.2) | **<0.001** |
| **MMEF_25-75_**  (L/s) | 2.6 (1.4) | 1.2  (0.6) | **<0.001** | 1.8 (1.2) | 0.8  (0.4) | **<0.001** | 1.8 (0.9) | 1.0  (0.5) | **<0.001** | 1.9 (0.9) | 0.7  (0.6) | **<0.001** |
| **FEV_3_/FEV_6_**  (%) | 95  (3) | 91  (2) | **<0.001** | 95  (4) | 91  (3) | **<0.001** | 94  (3) | 91  (2) | **<0.001** | 93  (3) | 87  (6) | **<0.001** |
| **TC 63%**  (s) | 0.54 (0.15) | 0.84  (0.11) | **<0.001** | 0.51 (0.2) | 0.86  (0.14) | **<0.001** | 0.66 (0.14) | 0.86  (0.12) | **<0.001** | 0.74 (0.10) | 1.25  (0.65) | **<0.001** |
