# Supplementary material for: Expiratory time constant patterns reveal obstructive lung mechanics in non-obstructive spirometry
Source: PLOS Digit Health. 2026 Aug 11;5(8):e0001653. doi: 10.1371/journal.pdig.0001653 (PMC13460623; doi:10.1371/journal.pdig.0001653)
Supplement: Supplementary file 3 [file pdig.0001653.s003.docx]

**S3 Table**

| **Table 3. Characteristics of subjects with and without isolated small-airway dysfunction among those with a preserved FEV₁/FVC ratio.** | | | |
| --- | --- | --- | --- |
| **Characteristic** | **Isolated SAD** (MMEF_25-75_ z < LLN)  **n = 1,839** | **Preserved**  (MMEF_25-75_ z ≥ LLN)  **n = 14,318** | ***p*** |
| **Obstructive TC pattern (n, %)** | 616 (33.5%) | 866 (6.0%) | <0.001 |
| **Sex Male (n, %)** | 760 (41.3%) | 6,359 (44.4%) | 0.01 |
| **Age (yrs)** | 56 (16) | 58 (15) | <0.001 |
| **Weight (kg)** | 85.1 (22.93) | 84.4 (19.46) | 0.19 |
| **Height (cm)** | 168.1 (10.25) | 169.0 (9.89) | <0.001 |
| **FEV₁ (L)** | 1.96 (0.79) | 2.74 (0.89) | <0.001 |
| **FEV₁ (z-score)** | −2.31 (1.04) | −0.55 (1.09) | <0.001 |
| **FVC (L)** | 2.72 (1.08) | 3.49 (1.11) | <0.001 |
| **FVC (z-score)** | −1.88 (1.46) | −0.47 (1.22) | <0.001 |
| **FEV₁/FVC (%)** | 71.9 (5.3) | 78.5 (6.1) | <0.001 |
| **FEV₁/FVC (z-score)** | −1.02 (0.59) | −0.03 (0.82) | <0.001 |
| **PEF (L/s)** | 4.92 (1.93) | 6.53 (2.11) | <0.001 |
| **PEF (z-score)** | −2.18 (1.33) | −0.67 (1.36) | <0.001 |
| **MEF₇₅ (L/s)** | 3.80 (1.46) | 5.75 (1.87) | <0.001 |
| **MEF₇₅ (z-score)** | −1.66 (0.71) | −0.40 (0.89) | <0.001 |
| **MEF₅₀ (L/s)** | 1.74 (0.77) | 3.26 (1.31) | <0.001 |
| **MEF₅₀ (z-score)** | −2.00 (0.33) | −0.71 (0.83) | <0.001 |
| **MEF₂₅ (L/s)** | 0.53 (0.32) | 1.04 (0.60) | <0.001 |
| **MEF₂₅ (z-score)** | −1.36 (0.53) | 0.00 (0.79) | <0.001 |
| **MMEF₂₅₋₇₅ (L/s)** | 1.35 (0.65) | 2.58 (1.11) | <0.001 |
| **MMEF₂₅₋₇₅ (z-score)** | −2.02 (0.34) | −0.36 (0.82) | <0.001 |
| **FEV₃/FEV₆ (%)** | 92.9 (2.9) | 95.1 (2.6) | <0.001 |
| **TC 63% (s)** | 0.73 (0.16) | 0.55 (0.14) | <0.001 |

Data are mean (SD) or n (%). Analysis restricted to subjects with a preserved FEV₁/FVC (z ≥ −1.645). Isolated small-airway dysfunction (SAD) was defined as an MMEF₂₅₋₇₅ z-score < LLN (<1.645). Continuous variables were compared by the Welch t-test and categorical variables by the chi-square test. z-scores were derived from GLI-2012 reference equations. LLN, lower limit of normal; SAD, small-airway dysfunction; TC_63_, expiratory time constant.
