# Supplementary material for: Expiratory time constant patterns reveal obstructive lung mechanics in non-obstructive spirometry
Source: PLOS Digit Health. 2026 Aug 11;5(8):e0001653. doi: 10.1371/journal.pdig.0001653 (PMC13460623; doi:10.1371/journal.pdig.0001653)
Supplement: Supplementary file 4 [file pdig.0001653.s004.docx]

**S1 Text: K-Means Clustering Methodology and Validation**

This supplement provides a detailed methodology and validation for applying K-means clustering to obtain spirometric trajectories using expiratory time constants (TC). The goal is to identify distinct obstructive spirometry patterns in a data-driven manner. The process includes data preparation, an objective determination of the optimal number of clusters, model execution, and results. The overview, including a step-by-step process for K-means clustering, is shown in Figure 1 below.

**Figure 1:** Overview of the data analysis pipeline, from initial data processing to model validation and interpretation. **(1)** Raw spirometry data were exported and cleaned from 19,937 flow-volume curves. **(2)** Key trajectory predictors (S1-S4) and their corresponding durations (S1d-S4d) were selected. **(3)** Predictor data were scaled to ensure equal contribution to the model. **(4)** Dimensionality was reduced using Principal Component Analysis (PCA), with the scree plot showing that the first two components explained over 84% of the variance. **(5)** The optimal number of clusters (*k*=2) was determined using statistical methods. **(6)** K-means clustering was trained exclusively on the normal spirometry subgroup. **(7)** The two discovered patterns were profiled by visualizing their mean trajectories, leading to their definition as 'normal' and 'obstructive'. **(8)** The trained model was applied to the entire dataset, with an alluvial plot visualizing the reclassification of subjects. **(9)** Model validation was performed by quantifying performance metrics such as accuracy and F1 score. **(10)** Final UMAP visualizations compare patient classifications based on standard spirometric categories (left), the new K-means identified patterns (middle), and a concordance plot highlighting reclassified individuals (right). **LLN** - Lower Limit of Normal; **PCA** - Principal Component Analysis; **UMAP** - Uniform Manifold Approximation and Projection, **S1d – S4d** – duration of S1 to S4.

**1. Data Preparation**

The full data processing is shown in Figure 1 in the main manuscript. Median Absolute Deviation (MAD) method was used to only outliers with values more than 8 x MADs from the median within each ATS category. To preserve generalizability, we removed only the most extreme outliers that might otherwise skew the cluster centroids, potentially impacting the cluster formation, losing 2% of the observations for S1 to S4 combined. Because the 8 × MAD removal was a pre-analysis safeguard rather than a deployment criterion, prospective patients with extreme values would be classified rather than excluded, and retaining them would not be expected to materially alter the pattern distributions.

**2. Predictor selection for K-means**

Knowing that patients classified as having obstruction (FEV_1_/FVC < LLN), we observed as ascending trajectory (i.e. S1 < S2 < S3 < S4 < S5, with their corresponding measured duration (S1 duration < S2 duration < S3 duration < S4 duration < S5 duration) – **Figure 2**.

**Figure 2** – Summary statistics presented as boxplots for S1 to S5 with a clear distinction between a normal pattern (blue arrow) and an ascending obstructive TC pattern (red arrow).

Based on this observation and physiological rationale (that with small airway disease, resistance progressively increases and therefore also consecutive TCs), we naturally observed two trajectories – ascending (occurring in patients with known obstruction and this trajectory was also suggested in patients with nonspecific FEV_1_ reduction) and relatively stable TCs presented in patients with normal or possible restriction groups (Figure 2).

**2.1. Removing the S5 TC from the model**

Plotting consecutive TCs vs their corresponding real measured times (each time corresponded to roughly 20% of analyzed volume portions of the descending flow/volume curve from PEF until the end of exhalation). Before executing K-means, we visualized the distances of individual points (TCs vs their real measured corresponding times) (Figure 3). This observation was important for selection of predictors for the K-means for three main reasons: **first** - a distant S5 would reduce the sensitivity and specificity of K-means to determine patterns correctly, **second** – with the last 20% (terminal) exhalation, the increasing TCs are already evident (meaning that increased Raw is well present) and the S5 would not be essential component for correct pattern recognition and **third** – the statistical variability of S5 (given the low flow conditions at terminal exhalation) was highest among all TCs, therefore introducing bias to the modelling. Based on the above, S1-S4 and their corresponding durations for the final model.

**Figure 3** – Shows compartmental TC (S1 to S5) on the y-axis against real measured time for each compartment on the x-axis (Expiratory time). Note the extreme distance of S5 from other TCs.

Following predictor selection. All predictors (S1-S4 and their corresponding measured durations) were standardized using z-score normalization to prevent a larger range, which would have dominated the clustering. By standardizing the data to have a mean of 0, each predictor was ensured to contribute equally to the cluster formation.

**3 and 4. Data scaling, dimensionality reduction, and principal component analysis (PCA)**

To visualize potential clusters and reduce noise, we used dimensionality reduction techniques on scaled data to visualize natural groupings before applying the K-means. Results of the cumulative variance can be seen on the scree plot (Figure 4) and natural clusters on the PCA plot below (Figure 5).

The scree plot (Figure 4) visualizes the percentage of variance explained by each principal component (PC). The first PC1 accounts for a significant portion of the variance (68.3%), followed by the second PC2 (14.5%). The "elbow" of the plot occurs after PC2, indicating that the first two components capture a substantial amount of the total information (82.8%) in the data.

**Figure 4 –** Scree plot shows that first two principal components explained nearly 83% of the data.

The PCA plot (Figure 5) shows the data projected onto the first two principal components, with points colored by their K-means cluster assignment. A clear separation between the two clusters is visible, confirming that the two well-defined patterns were identified. This visual evidence suggests the clusters are not random artifacts, but rather are supported by the data's inherent structure.

**Figure 5 –** Principal Component Analysis shows well separated regions between TC pattern datapoints.

**5. Justifying the Optimal Number of Clusters (K)**

To provide an objective but also physiologically sound number of clusters, an appropriate number was determined through a combination of statistical metrics and physiological reasoning. The Elbow and Silhouette methods were applied to a random sample (*n* = 6000) of the scaled data. As shown in the Elbow method plot, the optimal number of clusters, k, was identified as 2, as this is the point where the rate of decrease in the total within-cluster sum of squares sharply declines. This was supported by the Silhouette method, which showed a clear peak in the average silhouette width at *k* = 2, indicating the best-defined and most separated clusters at this number.

This data-driven decision to choose two clusters was further validated by the clinical context, as the primary goal of the study was to identify the presence of obstructive patterns within other non-obstructive spirometry (i.e., normal, possible restriction, and nonspecific FEV_1_ reduction), which naturally aligns with a binary classification (detecting obstruction against non-obstruction). Therefore, we finally selected 2 clusters for the K-means algorithm. (Increasing the cluster count to k = 3 did not add further information, yielding only a near-normal ‘straight’ pattern of non-prolonged TCs that did not capture obstructive pathology.)

**Figure 6 –** Elbow and Sihouette methods to determine appropriate number of clusters (patterns)

**6. Executing K-means Clustering**

Following the determination of an optimal cluster count (*k* = 2), the K-means algorithm was executed. The clustering model was first performed exclusively on the subset of the normal spirometry group (FEV_1_, FVC, and FEV_1_/FVC above LLN). This strategy establishes a baseline of expiratory mechanics and avoids biasing the model towards obstruction. The analysis was run 25 times using different random initial cluster centroids to mitigate the risk of converging on a suboptimal local minimum.  The best result, defined as having the lowest total within-cluster sum of squares, was selected.

**7. Interpreting and Characterizing Cluster Patterns**

After K-means execution, two clusters emerged and are shown in Figure 7. These were named normal pattern (shown in blue), showing the first decrease in TC from S1 to S2 and S3, increasing thereafter to S4. On the other hand, the ascending (obstructive) trajectory was labeled an obstructive pattern that aligns with clinical obstruction by LLN (dark red).

**Figure 7** – Presence of normal and obstructive patterns across all four spirometric categories. Normal pattern (blue) or obstructive pattern with progressively prolonging TCs (dark red).

**II. Model Validation**

**1. Internal Validation**

The stability and quality of the two clusters were first assessed internally. The Jaccard index, a measure of cluster stability, was high for both the obstructive pattern (0.989) and the normal pattern (0.994). These values approach excellent cluster stability, indicating the composition of each is highly reproducible and consistent.

The Average Silhouette Width of 0.31 suggests a reasonable degree of separation between the two clusters. The Davies-Bouldin Index could not be computed. Both methods suggest that, despite highly stable patterns, an overlap occurs, which is a potential limitation of the clustering method.

**2. External Concordance**

The clinical relevance of the K-means clusters was evaluated by comparing them against the established ATS spirometry categories, with "obstruction" serving as the clinical gold standard. The concordance metrics demonstrated a strong alignment between the data-driven patterns and clinical definitions. As reported in the main manuscript, 93% of subjects defined as normal by the lower limit of normal (LLN) had a normal pattern (TCs were relatively stable), and 91% classified as obstruction (LLN <- 1.645) had an obstructive pattern.

The model achieved an overall Accuracy of 0.9, indicating a high rate of correct classification. A Sensitivity of 0.913 confirms the model's effectiveness in identifying the significant majority of cases categorized as having obstruction by ATS/ERS classification. The Specificity was also high at 0.908, reflecting its ability to correctly identify non-obstructive patterns. The F1 Score of 0.792 provides a balanced measure of performance, confirming the model's robust predictive power.

**3. Comparative Robustness**

To ensure the findings were not an artifact of the K-means algorithm, the cluster assignments were compared against two alternative methods: **Hierarchical Clustering** (Figure 1) and **Partitioning Around Medoids (PAM)** (Figure 2)**.** The agreement was quantified using the Adjusted Rand Index (ARI), with scores ranging from -1 (random) to 1 (perfect agreement). The ARI scores of 0.307 (vs. Hierarchical) and 0.818 (vs. PAM) indicate substantial and strong agreement, respectively, meaning that identified spirometric patterns are intrinsic to the data and not an artifact of the chosen algorithm.

**Figure 1** shows a comparative analysis of K-means and Hierarchical clustering results projected onto a 2D UMAP space. Points are colored to represent cluster assignments: blue for the Normal pattern and red for the Obstructive pattern. Dashed ellipses visually delineate the 90% confidence intervals, illustrating the boundaries and degree of overlap between the two clusters.

**Figure 2** shows a comparative analysis of K-means and PAM clustering results projected onto a 2D UMAP space. Points are colored to represent cluster assignments: blue for the Normal pattern and red for the Obstructive pattern. Dashed ellipses visually delineate the 90% confidence intervals, illustrating the boundaries and degree of overlap between the two clusters.

**4. Summary**

In this analysis, the clustering successfully identified two stable patterns termed normal and obstructive, as shown by the high Jaccard and concordance scores. However, relatively low internal quality metrics provide a realistic interpretation, suggesting that both patterns exist on a continuous spectrum with an overlap, rather than being two perfectly separated groups in the data. A summary of all results is shown in **Table 1**.

**Table 1:** A summary of the validation and performance metrics for the K-means clustering. The table's structure includes assessments of internal stability and quality, external concordance against the gold standard (obstruction by LLN), and comparative robustness against alternative clustering methods. ARI: Adjusted Rand Index, PAM: Partitioning Around Medoids
